# The Immunomodulatory Small Molecule Imiquimod Induces Apoptosis in Devil Facial Tumour Cell Lines

**DOI:** 10.1371/journal.pone.0168068

**Published:** 2016-12-09

**Authors:** Amanda L. Patchett, Jocelyn M. Darby, Cesar Tovar, A. Bruce Lyons, Gregory M. Woods

**Affiliations:** 1 Menzies Institute for Medical Research, University of Tasmania, Hobart, TAS, Australia; 2 School of Medicine, University of Tasmania, Hobart, TAS, Australia; Columbia University, UNITED STATES

## Abstract

The survival of the Tasmanian devil (*Sarcophilus harrisii*) is threatened by devil facial tumour disease (DFTD). This transmissible cancer is usually fatal, and no successful treatments have been developed. In human studies, the small immunomodulatory molecule imiquimod is a successful immunotherapy, activating anti-tumour immunity via stimulation of toll-like receptor-7 (TLR7) signaling pathways. In addition, imiquimod is a potent inducer of apoptosis in human tumour cell lines via TLR7 independent mechanisms. Here we investigate the potential of imiquimod as a DFTD therapy through analysis of treated DFTD cell lines and Tasmanian devil fibroblasts. WST-8 proliferation assays and annexin V apoptosis assays were performed to monitor apoptosis, and changes to the expression of pro- and anti-apoptotic genes were analysed using qRT-PCR. Our results show that DFTD cell lines, but not Tasmanian devil fibroblasts, are sensitive to imiquimod-induced apoptosis in a time and concentration dependent manner. Induction of apoptosis was accompanied by down-regulation of the anti-apoptotic *BCL2* and *BCLX*_*L*_ genes, and up-regulation of the pro-apoptotic *BIM* gene. Continuous imiquimod treatment was required for these effects to occur. These results demonstrate that imiquimod can deregulate DFTD cell growth and survival in direct and targeted manner. *In vivo*, this may increase DFTD vulnerability to imiquimod-induced TLR7-mediated immune responses. Our findings have improved the current knowledge of imiquimod action in tumour cells for application to both DFTD and human cancer therapy.

## Introduction

The immunotherapeutic agent imiquimod (R-837) is a synthetic guanosine analogue recognised for its immune-stimulating capabilities. As an agonist of the single stranded RNA pattern recognition receptor toll-like receptor-7 (TLR7), imiquimod promotes potent anti-viral innate immunity and T helper-1 (T_H_1) type adaptive responses via activation of plasmacytoid dendritic cells, myeloid dendritic cells and macrophages [1–5]. TLR7 signaling in these cells stimulates nuclear factor kappa-B (NFκB) and interferon regulatory factor (IRF) pathways, leading to enhanced expression of genes required for antigen presentation and immune stimulation [1–4, 6]. As an immunotherapeutic agent, imiquimod has shown activity against epithelial and cutaneous pre-neoplastic, neoplastic and metastatic lesions [7]. Large-scale inflammatory infiltration and initiation of cytotoxicity are characteristic of a successful imiquimod treatment, and full lesion regression is common [8–11]. FDA approval was granted for the topical treatment of genital and perianal warts with imiquimod in 1997, and superficial basal cell carcinoma and actinic keratosis in 2004 [7].

The activation of tumour-specific T_H_1 responses during imiquimod immunotherapy is associated with tumour regression [8, 9, 12]. In addition, cell culture studies have demonstrated direct activation of tumour cell apoptosis and autophagy in response to imiquimod treatment [13–17]. Imiquimod exerts these effects in a tumour specific manner, independently of both TLR7 signaling and immune cell function [13, 15, 18, 19]. Pro- and anti-apoptotic proteins, such as Noxa, BCL2 and MCL1, are involved in the regulation of these apoptotic pathways, and more recently both oxidative and endoplasmic reticulum (ER) stress pathways have also been implicated [14, 18–21]. The impact of these molecular changes on the efficacy of imiquimod immunotherapy remains undefined.

Devil facial tumour disease (DFTD) describes two genetically distinct transmissible tumours threatening wild populations of Tasmania’s largest extant marsupial, the Tasmanian devil (*Sarcophilus harrisii*) [22]. Initially observed in 1996, the first of the tumours (DFT1) is of Schwann cell origin, and has caused severe population declines across most of the devil’s natural habitat [23–25]. In comparison, the newer tumour (DFT2) was discovered in 2014, is of unknown origin, and is currently localised to a small region of South-Eastern Tasmania [22]. Both DFT1 and DFT2 are somatic cell lineages transmitted as allografts due to a lack of recognition by the devil’s immune system [22, 26, 27]. With little natural immunity against the tumour cells, developing successful therapies and vaccinations against these cancers has proven to be challenging [27–29]. An immunotherapy against DFTD, although challenging to distribute among a wild devil population, could break immune tolerance against the tumour and may promote long-term immunity against the disease. Investigation of these mechanisms may also reveal new techniques for the development of a prophylactic DFTD vaccine.

Previously, we demonstrated that Tasmanian devil mononuclear cells (MNCs) express functional TLR7. As with many human tumour cell lines, we also found that DFT1 cells in culture underwent apoptosis after imiquimod treatment [30]. Taken together these data suggest that imiquimod has potential as a DFTD immunotherapy. Here we report a more detailed analysis of the response of DFTD cultures to imiquimod treatment. These data will enable a greater understanding of imiquimod induced tumour cell death for application to both DFTD and human cancer therapy.

## Methods

### Cell culture

DFT1 cell lines C5065, 1426, 4906 and ½Pea were provided by A-M. Pearse and K. Swift of the Tasmanian Department of Primary Industries, Parks, Water and Environment (DPIPWE). These cell lines were previously established from DFT1 biopsies obtained under the approval of the Animal Ethics Committee of the Tasmanian Parks and Wildlife Service (permit numbers 33/2004-5 and 32/2005-6). The DFT2 cell line RV was established from a DFT2 biopsy and the fibroblast cell line TD344 was established from a Tasmanian devil tissue biopsy (permit numbers A0012513 and A0014976). Stocks stored in liquid nitrogen were thawed when required and cultured in RPMI medium (GIBCO, New York, USA) supplemented with 10% heat-inactivated foetal calf serum (FCS) (GIBCO, New York, USA), 1% GlutaMAX^TM^ (GIBCO, New York, USA) and 1% Antibiotic Antimycotic (GIBCO, New York, USA) (RPMI/10FCS). DFT2 and fibroblast cell lines were cultured in RPMI/10FCS supplemented with 10% AminoMAX^TM^-II Complete Medium (GIBCO, New York, USA). The cultures were maintained at 35°C in a humidified 5% CO_2_ incubator. When required for experimentation, DFTD cells were gently flushed from their culture surface using RPMI/10FCS. Adherent fibroblasts were washed in phosphate buffered saline (PBS) and removed from their surface with 0.025% Trypsin-EDTA (GIBCO, New York, USA) at 37°C for 2 minutes. RPMI/10FCS was added to inhibit the trypsin. Cells were pelleted by centrifugation at 500 g for 5 min. Cell viability counts were performed on an improved neubauer haemocytometer by trypan blue dye exclusion.

### Imiquimod stimulation

Imiquimod (AdipoGen, San Diego, USA) was dissolved in dimethyl sulfoxide (DMSO) and diluted to working concentrations with sterilised water or RPMI/10FCS. Unless otherwise stated, cell cultures were stimulated with imiquimod at 60 μg/ml (0.5% DMSO). Vehicle controls were treated with DMSO at concentrations equivalent to experimental cultures. Stimulated cultures were maintained at 35°C in a humidified 5% CO_2_ incubator for the treatment duration. Cell lines ‘pulsed’ with imiquimod were treated for 24 or 48 h, washed three times in sterile PBS, and re-cultured in RPMI/10FCS without imiquimod treatment for the remainder of the experiment (pulsed cells).

### Cell proliferation analysis (WST-8 Assay)

The DFT1 cell lines C5065, 1426 and 4906, the DFT2 cell line RV and the non-transformed fibroblast cell line TD344 were seeded into flat-bottom 96-well cell culture plates at a concentration of 1x10^5^ cells/ml in 100 μl of RPMI/10FCS. Cells were treated in triplicate with imiquimod at 7.5, 15.0, 30.0, 60.0 or 120.0 μg/ml for 0, 12, 24, 48, 72, 96 or 120 h. Relative cell number in culture was analysed using the Cell Counting Kit-8 (WST-8) (Sigma-Aldrich, St Louis, USA) according to the manufacturer’s instructions. Absorbance was measured at OD_570_-OD_650_ using the SpectraMax Plus 384 plate reader (Molecular Devices, Sunnyvale, USA). Baseline metabolic rate was determined by absorbance at the 0 h time point. Changes to cell number were calculated relative to untreated cultures.

### Annexin V cell death assay

The DFT1 cell line C5065 and DFT2 cell line RV were seeded into 24-well cell culture plates at a concentration of 1x10^5^ cells/ml in 500 μl of RPMI/10FCS. Cells were treated with imiquimod for 8, 16, 24, 48, 72, 96 or 120 h. Analysis of cell death was performed using the Annexin V cell death assay (BD Pharmingen, San Diego, USA), as previously described [30]. Samples were analysed on the FACSCanto^TM^ II Flow Cytometer (BD Biosciences, San Jose, USA). Kaluza^®^ Flow Analysis Software (Beckman Coulter, Pasadena, USA) was used to calculate the percentage of cells positive for annexin V and propidium iodide (PI). Viable cells were negative for both annexin V and PI staining, necrotic cells were positive for PI staining, early apoptotic cells were positive for annexin V staining, and late apoptotic cells were positive for both annexin V and PI staining.

### Cell cycle analysis

The DFT1 cell line C5065, the DFT2 cell line RV and the fibroblast cell line TD344 were seeded into 24-well culture plates at a concentration of 1x10^5^ cells/ml in 500 μl of RPMI/10FCS. Cells were treated with imiquimod for 48 h. Cell cycle analysis was performed according to the methods of Riccardi and Nicoletti [31], as previously described [30]. PI staining was analysed by flow cytometry using the FACSCanto^TM^ II flow cytometer. The Kaluza^®^ Flow Analysis Software was used to compile histograms and measure hypodiploid and diploid peaks. A hypodiploid peak represents DNA fragmentation and suggests that apoptotic cells are present.

### Tasmanian devils and biological samples

Primary tissue samples were obtained from Tasmanian devils with the approval of the University of Tasmania Animal Ethics Committee (permit numbers A0012513 and A0014976). Devils were anaesthetized with isofluorane gas in oxygen at a rate of 2 L/minute and approximately 10 ml of blood was obtained from the jugular vein as previously described [32]. Blood was transported in lithium heparin anticoagulant tubes (Greiner Bio-One, Kremsmunster, Austria) at room temperature and processed within 24 h of collection. Peripheral blood mononuclear cells (PBMNCs) were isolated using density gradient centrifugation on Histopaque-1077 (Sigma-Aldrich, St Louis, USA), according to the manufacturer’s instructions. DFTD biopsies were collected from diseased wild devils using a 4 mm disposable biopsy punch (Kai Medical, Solingen, Germany). Peripheral nerve samples were collected from the brachial plexus or sciatic nerve of euthanised Tasmanian devils. Tumour biopsies and peripheral nerve samples were immediately placed in RNAlater and transported on ice. Samples were homogenized in TriReagent (Sigma-Aldrich, St Louis, USA) with a Mini Beadbeater-24 (BioSpec Products, Bartlesville, USA) and 2.0 mm Zirconia Beads (BioSpec Products, Bartlesville, USA).

### RNA extraction and cDNA preparation

Cell lines and biological samples were lysed in 1 ml of TriReagent and RNA was extracted according to the manufacturer’s instructions. RNA was suspended in TE buffer (pH 8.0) (Ambion, Austin, USA) and DNase treated with the TURBO DNA-*free*^TM^ kit (Ambion, Austin, USA). The concentration and purity of the RNA was assessed using a NanoDrop 1000 Spectrophotometer (NanoDrop Technologies, Wilmington, USA). Samples with an A260/A280 ratio of greater than 1.8 were selected and 0.5–1.0 μg of total RNA was reverse transcribed to cDNA using the GoScript^TM^ Reverse Transcription System (Promega, Madison, USA). A non-reverse transcribed control (no-RT control) was included to identify the presence of any contaminating genomic DNA.

### Quantitative reverse transcriptase polymerase chain reaction (qRT-PCR)

Primer sequences were designed for target genes identified in the Tasmanian devil reference genome Devil7.0 assembly GCA_000189315.1, using the NCBI PrimerBLAST tool (Table 1). Primers were synthesized by GeneWorks (Adelaide, Australia). qRT-PCR was performed using the LightCycler^®^ 480 SYBR Green I Master (Roche, Indianapolis, USA) and LightCycler^®^ 480 (Roche, Indianapolis, USA), as previously described [30]. Expression of *18S rRNA* was measured as reference gene, and no-template controls and no-RT controls were included in all analyses. Standard curves were performed for validation of reaction efficiency, and the comparative Ct method was used for calculation of expression fold change [33]. Statistical significance was calculated from log_2_-converted values using a repeated measures one-way ANOVA and a Dunnett’s multiple comparisons test. Statistical significance was defined as * = <0.05, ** = <0.01, *** = <0.001.

**Table 1:** Primers designed for RT-PCR.

Gene	Forward Primer (5'-3')	Reverse Primer (5'-3')
*18S rRNA*	AGCGGCTGAAGAAGATACGG	TTGGACACACCCACAGTACG
*A20*	TAACCAGAAAGAGCAGGACCAC	ATCAGACAGAGCTCACAAGGTG
*BCL2*	GCGGATTGTGGCCTTCTTTG	AGTCATCCACAGGGCTATGC
*BCLXL*	AGAATCCACCCTCGGAAACC	CAGGAATGGGCTGATCCAGT
*MCL1*	AGTTGTACGGGCAGTCCTTG	CCCCGTCACTGAACACATGA
*BAD*	ATGAGCGACGAGTTCCACTG	CAAATTCCGCCCGAACCAAG
*BAK*	CTACCGGCTGGCACTATATGTT	AATGGAGTTGTTGGAGAGGTCC
*BID*	CAGCCCAGCTTGGTGGATAA	GGATGGGGCATGGGTCATAA
*BIM*	CGTTTGCTACCAGATCCCCA	CACAACTCATAGGCGCTGGA
*TLR7*	CAGGACCAGGAGCACACAAA	TCTGGTGAAACTAGGCGCTG

### Agarose gel electrophoresis

qPCR products were visualized on 1.5% agarose gels by electrophoresis for 40 min at 100 V. A Quick-Load 100 base-pair DNA ladder (New England Biolabs, Ipswich, USA) was included for determination of PCR product length. Photographs of gels were taken with a Carestream Image Station 4000 MM (Carestream, Toronto, Canada).

## Results

### Imiquimod reduces DFT1 and DFT2 cell number in a time and concentration dependent manner

**Fig 1:**
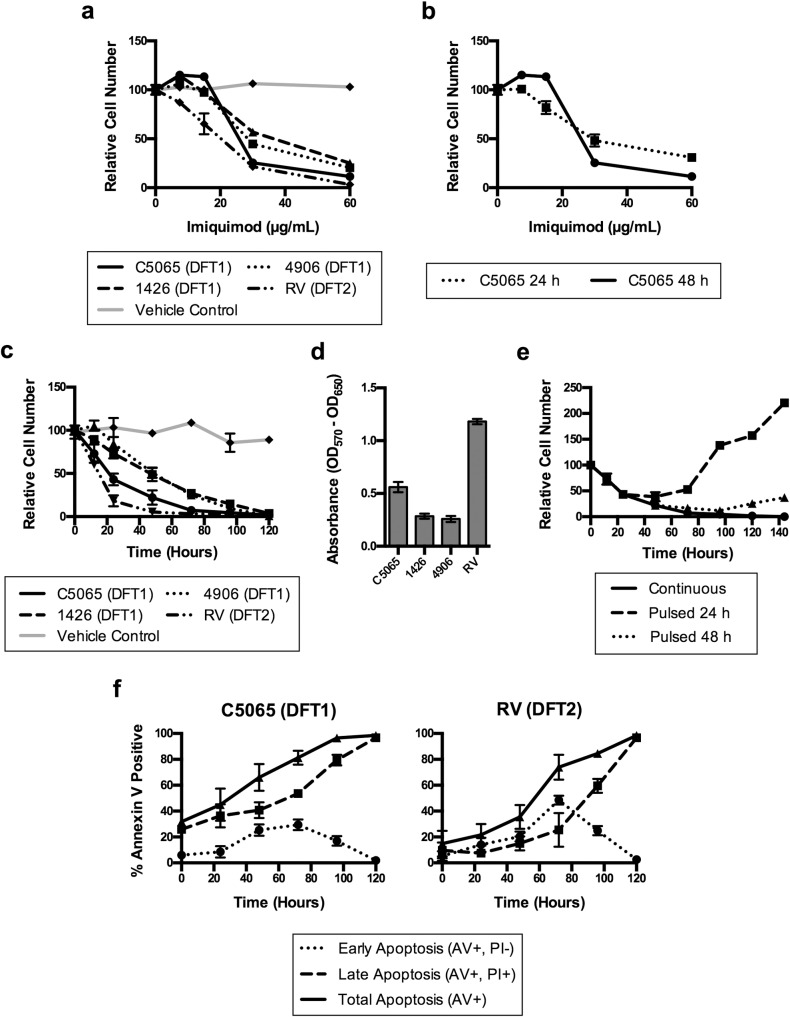
Imiquimod decreases DFTD cell number in culture in a concentration and time dependent manner. (a) Modulation of relative cell number in culture by imiquimod treatment was measured using WST-8 assays in DFT1 cell cultures (C5065, 1416, 4906) and a DFT2 cell culture (RV) treated at the indicated imiquimod concentrations. Cultures were treated with 0.5% DMSO as a vehicle control, which had no effect. Mean percent relative cell number was calculated by comparison to untreated cultures (b) C5065 DFT1 cultures were treated for 24 or 48 h at the indicated imiquimod concentrations and mean percent relative cell number was calculated by comparison to untreated cultures using WST-8 assays. (c) DFT1 cultures (C5065, 1416, 4906) and a DFT2 culture (RV) were treated with imiquimod (60 μg/ml) over a 120 h time course and mean percent relative cell number was calculated by comparison to untreated cultures using WST-8 assays. Cultures were treated with 0.5% DMSO as a vehicle control, which had no effect. (d) Baseline metabolic rate of C5065, 1426, 4906 and RV cultures was determined by WST-8 absorbance measures from untreated cells. (e) C5065 cells treated with imiquimod for 24 or 48 h were washed to remove imiquimod and recovered in culture with no treatment. Mean percent relative cell number was calculated and compared with a continuously treated culture over 140 h using WST-8 assays. (f) Induction of apoptosis by imiquimod treatment was measured in C5065 and RV cell lines over a 120 h time course using Annexin V assays. Annexin V (AV) binding identified cells undergoing early and late apoptosis, while PI staining identified cells in late apoptosis only. All results are the mean and standard error of three replicates.

Previous studies show that the immunotherapeutic agent imiquimod induces apoptosis in a range of tumour cell lines, including the DFT1 cell line C5065 [15, 16, 30, 34]. To investigate the effects of imiquimod more thoroughly, we analysed DFT1 cell lines (C5065, 1426, 4906) and a DFT2 cell line (RV) after treatment in culture. We first examined the effects of imiquimod using a WST-8 proliferation assay, which measures metabolic activity as an approximation of relative cell number in culture. Our data showed that continuous imiquimod treatment decreased DFT1 and DFT2 cell number in a concentration and time dependent manner. High imiquimod concentrations were required to reduce DFTD cell number, with lower concentrations increasing cell number in several cultures (Fig 1A). This increase is likely an artifact of decreased metabolic activity in untreated cultures as a result of overcrowding, as it was not detected at an end-point of 24 h (Fig 1B). At a high imiquimod concentration (60 μg/ml), cell number decreased gradually over a 120 h time period (Fig 1C). All DFTD cultures were sensitive to this change, but responded at different rates, with the DFT2 cell line RV demonstrating the most rapid reduction. The rates at which DFTD cell lines responded to imiquimod treatment correlated with their baseline metabolic rate, which was highest in RV cultures, moderate in C5065 cultures and lowest in 1426 and 4906 cultures (Fig 1D). Reductions in cell number were not maintained after pulsing of DFT1 cells with high concentrations of imiquimod for 24 or 48 h, suggesting that continuous imiquimod treatment is required for these effects to occur (Fig 1E).

We next determined whether induction of apoptosis could account for reduced DFTD cell proliferation in imiquimod treated cultures. We used annexin V and PI staining to measure the percentage of early, late and total apoptotic cells in the more rapidly responding cell lines C5065 (DFT1) and RV (DFT2). Our data show a time dependent increase in the percentage of DFTD cells undergoing apoptosis after imiquimod treatment (Fig 1F). In both cell lines, the percentage of cells entering apoptotic pathways peaked at 72 h, with complete apoptosis occurring after 120 h. These data suggest that imiquimod inhibits cell proliferation and subsequently activates apoptosis in imiquimod treated DFTD cell lines.

### Imiquimod regulates the expression of anti-apoptotic genes in DFTD cell lines

**Fig 2:**
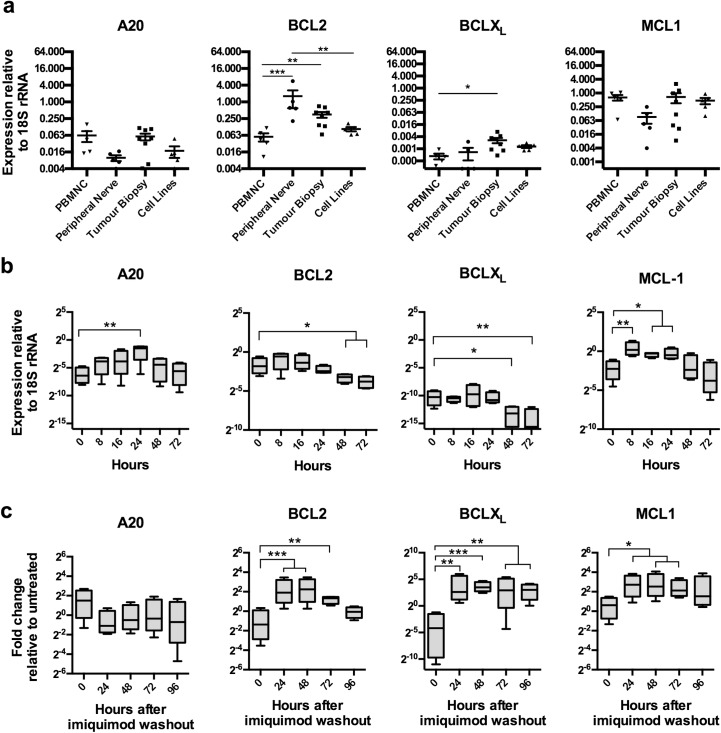
Imiquimod modulates the expression of anti-apoptotic genes in DFTD cell lines. (a) Baseline expression. Anti-apoptotic gene expression was analysed in RNA samples from PBMNC isolates, peripheral nerve samples, primary DFTD biopsies and DFTD cell lines using qRT-PCR. Each marker represents gene expression level in an individual biological sample or cell line. Results are displayed as the mean and standard error of expression relative to *18S rRNA*. (b) During treatment. Anti-apoptotic gene expression was measured by qRT-PCR in the DFT1 cell lines C5065, 4906 and 1426, and the DFT2 cell line RV, after continuous treatment with imiquimod (60 μg/ml) over a 72 h time course. Box-and-whisker plots represent the minimum and maximum expression values relative to *18S rRNA*, with the upper quartile, median and lower quartile expression values indicated by the box. (c) After treatment. Anti-apoptotic gene expression was measured by qRT-PCR in the cell lines C5065, 4906, 1426 and RV that were washed after treatment with imiquimod (60 μg/ml) for 48 h, and recovered for 96 h. Box-and-whisker plots represent the minimum and maximum fold-changes relative to untreated samples, with the upper quartile, median and lower quartile expression values indicated by the box. Statistical significance compared to the 0 h sample is defined as *<0.05, **<0.01, ***<0.001.

As with other inducers of apoptosis, imiquimod activates apoptotic pathways in tumour cell lines via regulation of anti-apoptotic genes [15, 20, 35, 36]. To determine whether this occurs in treated DFTD cultures, we measured the expression of the anti-apoptotic genes *BCL2*, *BCLX*_*L*_, *MCL1* and *A20* at various time points after imiquimod treatment using qRT-PCR. As DFT1 and DFT2 cell lines responded to imiquimod treatment in a similar manner, they were grouped together for this analysis. Anti-apoptotic genes had not previously been investigated in the Tasmanian devil, so we first measured their baseline expression in PBMNCs and peripheral nerve, a source of Schwann cells. We compared expression levels to primary DFTD biopsies and DFT1 (C5065, 1426, 4906, ½Pea) and DFT2 (RV) cell lines. Relative to *18S rRNA*, all anti-apoptotic genes were expressed at low to moderate levels, and were varied across the tested tissues (Fig 2A). Expression of anti-apoptotic genes in DFTD primary tumours and cell lines was within the range of PBMNC and peripheral nerve samples, suggesting that these genes are not overexpressed beyond normal levels. In response to imiquimod treatment, *A20* and *MCL1* were significantly up regulated at 24 h, before decreasing as the cells entered apoptosis (Fig 2B). The peak in expression occurred earlier for *MCL1* when compared to *A20*, a negative regulator of extrinsic apoptosis. Only *BCL2* and *BCLX*_*L*_ were down regulated significantly over the 72 h period. Continuous imiquimod treatment was required for this to occur, as DFTD cultures treated for only 48 h with imiquimod recovered expression *BCL2*, *BCLX*_*L*_ *and MCL1*, but not A20, to levels significantly higher than untreated cultures after the treatment was removed (Fig 2C). Together these data suggest that anti-apoptotic genes play a role in the regulation of apoptosis in DFTD cells after imiquimod treatment.

### Imiquimod regulates the expression of pro-apoptotic genes in DFTD cell lines

**Fig 3:**
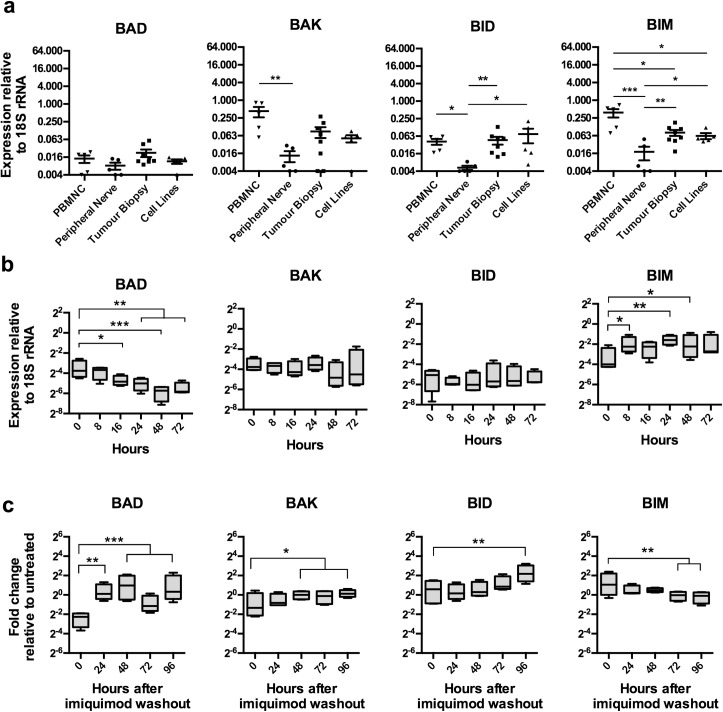
Imiquimod modulates the expression of pro-apoptotic genes in DFTD cell lines. (a) Baseline expression. Pro-apoptotic gene expression was analysed in RNA samples from PBMNC isolates, peripheral nerve samples, primary DFTD biopsies and DFTD cell lines using qRT-PCR. Each marker represents gene expression level in an individual biological sample or cell line. Results are displayed as the mean and standard error of expression relative to *18S rRNA* (b) During treatment. Pro-apoptotic gene expression was measured by qRT-PCR in the DFT1 cell lines C5065, 4906 and 1426, and the DFT2 cell line RV, after continuous treatment with imiquimod (60 μg/ml) over a 72 h time course. Box-and-whisker plots represent the minimum and maximum expression values relative to *18S rRNA*, with the upper quartile, median and lower quartile expression values indicated by the box. (c) After treatment. Pro-apoptotic gene expression was measured by qRT-PCR in the cell lines C5065, 4906, 1426 and RV that were washed after treatment with imiquimod (60 μg/ml) for 48 h, and recovered for 96 h. Box-and-whisker plots represent the minimum and maximum fold-changes relative to untreated samples, with the upper quartile, median and lower quartile expression values indicated by the box. Statistical significance compared to the 0 h sample is defined as *<0.05, **<0.01, ***<0.001.

Pro-apoptotic proteins of the BCL2 family have also been implicated in the initiation of tumour cell death after imiquimod treatment [21]. Genes for *BAD*, *BID*, *BIM*, *BAK* and *BOK* are annotated in the Tasmanian devil genome and were analysed for expression as described above. As expression of *BOK* was not detectable in DFTD cultures at any stage of imiquimod treatment, this gene was not included in the analysis. Relative to *18S rRNA*, all pro-apoptotic genes were expressed at low to moderate levels, and were varied across the tested tissues (Fig 3A). Expression of pro-apoptotic genes in DFTD primary tumours and cell lines was within the range of both PBMNC and peripheral nerve samples, suggesting that these genes are not abnormally expressed beyond normal levels. In response to imiquimod treatment, expression of the pro-apoptotic genes *BAK* and *BID* remained unchanged, while expression of *BAD* significantly decreased and *BIM* significantly increased over the 72 h treatment period (Fig 3B). Cultures treated with imiquimod for only 48 h demonstrated increased expression of *BAD*, *BAK and BID*, and decreased expression of *BIM* after the treatment was removed. These results suggest that regulation of pro-apoptotic genes plays a role in apoptosis activated in DFTD cells after imiquimod treatment.

### Expression of TLR7 is absent from DFT1 cell lines

**Fig 4:**
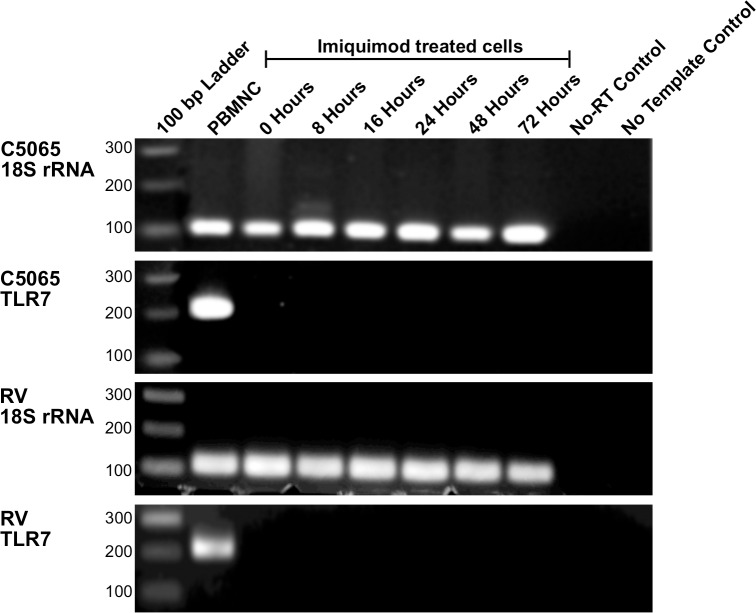
DFTD cell lines do not express TLR7 after imiquimod treatment. TLR7 expression was measured in the DFT1 cell line C5065 and DFT2 cell line RV treated with imiquimod (60 μg/ml) over a 72 h time course. PBMNCs were analysed as a positive control. Extracted RNA was analysed for expression of TLR7 using qRT-PCR. *18S rRNA* was amplified as a reference gene. PCR products were visualized on a 2% agarose gel with a 100 bp ladder for size comparison.

Although imiquimod is an agonist of TLR7, previous studies have shown that it can exert effects in tumour cells via TLR7-independent mechanisms [13, 18, 37, 38]. To determine whether this is the case in DFTD, we examined TLR7 expression in DFT1 and DFT2 cell lines. qRT-PCR products from untreated and imiquimod treated cells were visualized by agarose gel electrophoresis (Fig 4). We found that neither DFT1 nor DFT2 cell lines express detectable levels of TLR7 prior to or during imiquimod treatment. These results indicate that TLR7 is absent from DFTD cell lines and does not play a role in the activation of apoptotic pathways after imiquimod treatment.

### Imiquimod activates apoptosis in DFTD cells, but not Tasmanian devil fibroblasts

**Fig 5:**
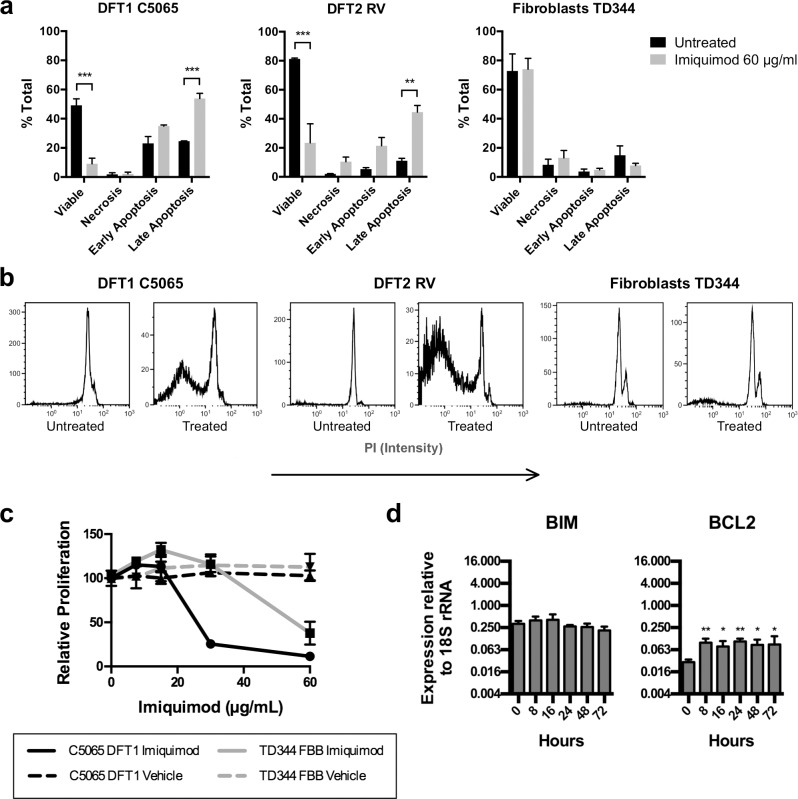
Apoptotic pathways are not activated in Tasmanian devil fibroblasts after imiquimod treatment. Cellular responses to imiquimod treatment were measured in the DFT1 cell line C5065, DFT2 cell line RV and non-transformed fibroblast cell line TD344. (a) Induction of apoptosis by imquimod was measured through detection of annexin V binding (early and late apoptosis) and PI staining (late apoptosis and necrosis) after 48 h of imiquimod treatment. (b) DNA fragmentation was measured in fixed cells by flow cytometry analysis of total DNA staining with PI. A hypodiploid peak represents fragmented DNA and indicates that cells are apoptotic. (c) Modulation of cell number in C5065 and TD344 cultures by imiquimod treatment was measured using a WST-8 assay. Mean percent relative cell number was calculated by comparison to untreated cultures. Cells were treated with 0.5% DMSO as a vehicle control, which had no effect. (d) Expression of pro-apoptotic *BIM* and anti-apoptotic *BCL2* was anaysed in RNA samples from treated fibroblasts using qRT-PCR. Gene expression levels were measured relative to *18S rRNA*. All results are displayed as the mean and standard error of three replicates. Statistical significance is defined as *<0.05, **<0.01, ***<0.001.

Previous investigation suggests that imiquimod-induced apoptosis occurs only in tumour cells [15]. To explore this further, a non-transformed Tasmanian devil fibroblast cell line (TD344) with no detectable expression of TLR7 was treated with imiquimod. We compared the responses of the fibroblast cell line with DFTD cultures using apoptosis, proliferation and gene expression assays. Our data show that DFT1 and DFT2 cells, but not fibroblasts, undergo apoptosis after treatment with imiquimod (Fig 5). Co-binding of annexin V and PI demonstrates a significant difference in the percentage of non-viable cells between untreated and imiquimod-treated samples for DFT1 and DFT2 cell lines (Fig 5A). This trend is not replicated in fibroblast cultures, suggesting that apoptotic pathways are not activated in these cells. To confirm this finding we analysed DNA fragmentation using cell cycle analysis. We detected large hypodiploid (sub-G1) peaks in imiquimod treated DFT1 and DFT2 cultures, but not fibroblast cultures, confirming that imiquimod does not activate apoptosis in these cells (Fig 5B). To understand whether imiquimod had any effect on the growth of Tasmanian devil fibroblasts, WST-8 assays were performed. High imiquimod concentrations (60 μg/ml) reduced cell number in fibroblast cultures by around 50% after 48 h of treatment, suggesting that imiquimod suppresses their growth without activating apoptotic pathways (Fig 5C). Expression analysis of the pro-apoptotic gene *BIM* and anti-apoptotic gene *BCL2* over a 72 h treatment period revealed that *BCL2* expression was heightened for the treatment duration, while *BIM* remained unchanged (Fig 5D). As we previously demonstrated that *BCL2* was down regulated and *BIM* up regulated by imiquimod treatment in DFTD cells, these results suggest that differential regulation of pro- and anti- apoptotic proteins plays a role in preventing imiquimod-induced apoptosis in Tasmanian devil fibroblasts.

## Discussion

DFTD continues to threaten the survival of the Tasmanian devil. Efforts to develop a successful prophylactic vaccine against the disease have produced mixed results, with DFTD-specific responses generating only short-term disease protection [29]. A successful DFTD therapy could provide an additional conservation strategy for diseased devils captured during routine trapping expeditions. Only two previous studies have investigated the use of therapeutic agents in DFTD. These studies analysed the chemotherapeutic drugs doxorubicin, carboplatin and vincristine, and found none of these to be effective in the treatment of the DFTD tumour [39, 40]. As DFTD is transmitted as a foreign allograft [26, 22], immunotherapy may be a more effective treatment strategy, with stimulation of an anti-tumour immune response perhaps sufficient to break immune tolerance and promote regression of the DFTD allograft.

The immunotherapeutic agent imiquimod activates TLR7-dependent immune responses, resulting in anti-tumour cytotoxicity and tumour regression in human cancers [8–11]. In the Tasmanian devil, if imiquimod could produce an allogeneic response against DFTD with minimal treatments, it would be feasible to opportunistically treat wild diseased devils captured during routine trapping expeditions with this drug. In human studies imiquimod has also been shown to directly modulate tumour cells via activation of apoptotic pathways independently of TLR7 [13–16, 21, 41]. *In vivo*, the growth of xenograft tumours in nude mice was completely suppressed by imiquimod therapy, highlighting roles for imiquimod-induced effects in the absence of an adaptive response [41]. As such, investigation into the mechanisms of imiquimod action in DFTD cells could also reveal new strategies for targeting this aggressive cancer, and could contribute to the development of a successful prophylactic DFTD vaccine.

To gain a better understanding of the effects of imiquimod in DFTD, we performed *in vitro* studies, investigating changes to DFT1 and DFT2 tumour cell lines after treatment. Although DFT1 and DFT2 tumours arose independently [22], both underwent time dependent growth inhibition and activation of apoptosis after imiquimod treatment. Consistent with previous *in vitro* studies, high concentrations were required for the initiation of these effects [13–15, 21]. These findings reflect the high amounts of imiquimod required for the immunotherapy of superficial basal carcinoma in humans, with FDA guidelines suggesting topical application of 0.5–2 mg of imiquimod almost daily [42]. It has been suggested that apoptotic pathways in imiquimod treated tumour cells are activated as a result of ROS accumulation, which enhances ASK-1 stimulation of JNK and p38 apoptotic pathways [18, 21, 37, 43–45]. Imiquimod-induced ROS production may occur as a result of calcium induced deregulation of oxidative phosphorylation [21], and rapid accumulation is likely due to the elevated metabolic activity of tumour cells. This hypothesis is supported by our results, which show a faster response to imiquimod treatment in cell lines with a higher baseline metabolic rate.

The regulation of pro- and anti-apoptotic proteins in imiquimod treated tumour cells has been well established, with several studies measuring changes to BCL2, MCL1, A20 and Noxa after treatment [13–15, 20, 21, 35, 41]. Recently, El-Khattouti et al. suggested that localization of pro-apoptotic Noxa to mitochondria is required for a loss of mitochondrial membrane potential, cytochrome C release and subsequent apoptosis of imiquimod-treated melanoma cell lines. Activity of Noxa was controlled through ER-stress mediated activation of PERK signaling and ROS-mediated JNK activation [21]. We were unable to measure Noxa expression due to incomplete annotation of the Tasmanian devil genome, but we did detect up-regulation of *BIM*, a pro-apoptotic protein also activated through ER-stress pathways [46]. This suggests that similar molecular changes are triggered by imiquimod in DFTD cells. El-Khattouti et al. hypothesized that ER stress pathways are mediated through TLR7 activation [21], however we could not detect any expression of TLR7 in our cell lines prior to or after imiquimod treatment. As previous studies have also reported TLR7 independent activation of apoptosis in imiquimod treated tumour cell lines [13, 15, 18, 19, 37], we suggest imiquimod induces ER stress via alternative mechanisms.

The anti-apoptotic genes *BCL2*, *MCL1* and *A20* are regulated in tumour cells by imiquimod, allowing the onset of apoptotic pathways [13, 15, 20, 35]. We detected significant down-regulation of genes encoding BCL2, but not MCL1 or A20. It remains possible that *A20* and *MCL2* were further down regulated after 72 h of imiquimod treatment, but we were unable to extract quality RNA to test this due to low cell viability. MCL1 and A20 are involved in suppression of apoptosis through interactions with Noxa and BIM, and degradation of ASK1, respectively [47, 48]. We detected transient up regulation of these genes immediately after treatment, suggesting that there may be early activation of pathways protective against Noxa and BIM-mediated apoptosis in response to imiquimod. This could explain both the prolonged survival of imiquimod treated tumour cells in culture for 96 to 120 h, and the full recovery of imiquimod pulsed cultures. *In vivo*, frequent application of imiquimod may be required to overcome these pathways and induce tumour cell apoptosis. Inhibitors of protective pathways may also have therapeutic benefit during these treatments by potentiating imiquimod-induced effects. Human cancer therapies utilize daily topical treatments with imiquimod as a normal practice [49], but a repetitive protocol of treatment is less feasible in a wild species such as the Tasmanian devil. *In vivo* studies are required to determine whether rejection of the DFTD allograft is possible in the absence of repetitive treatments.

Although imiquimod is a potent inducer of apoptosis in tumour cells, these effects do not extend to normal tissues. *In vivo*, this may restrict imiquimod-induced effects to the tumour site, preventing substantial damage of surrounding tissue. Our findings align with previous research where primary keratinocyte cultures were resistant to imiquimod-induced effects, but treated tumour cells and transformed keratinocyte cultures underwent apoptosis [15]. The uncontrolled nature of tumour cell growth and metabolism may allow imiquimod-induced cellular stressors such as ROS to accumulate at a greater rate in these cells, overwhelming defenses against cellular damage. In addition, neoplastic cells cope with ROS and other stressors at heightened baseline levels relative to normal cells, suggesting that their coping mechanisms may be more easily exhausted by additional stressors [50, 51]. Although imiquimod did not activate apoptosis of Tasmanian devil fibroblasts, there was a decrease in cell number after treatment, suggesting an inhibitory effect on growth. Cell cycle arrest has been documented in response to imiquimod treatment, and could allow resolution of cellular stress in normal cells [52]. Aberrations to these mechanisms may lead to the activation of apoptotic pathways in imiquimod treated tumour cells.

In summary, we have demonstrated that imiquimod selectively induces apoptosis in DFT1 and DFT2 cell lines. Our results have highlighted the importance of continuous imiquimod treatment for activation of these pathways, which may have implications for its therapeutic use in both DFTD and human cancers. As a DFTD immunotherapy, imiquimod has potential to activate TLR7 mediated immune responses, while deregulating tumour growth and survival in a direct and targeted manner. Together this suggests that imiquimod is suitable for DFTD therapy, warranting further investigation into strategies for its use in the Tasmanian devil.
